# Portals of Change: How Patient Portals Will Ultimately Work for Safety Net Populations

**DOI:** 10.2196/16835

**Published:** 2020-10-23

**Authors:** Alejandra Casillas, Anshu Abhat, Anish Mahajan, Gerardo Moreno, Arleen F Brown, Sara Simmons, Peter Szilagyi

**Affiliations:** 1 Division of General Internal Medicine and Health Services Research David Geffen School of Medicine Los Angeles, CA United States; 2 Los Angeles County Department of Health Services Los Angeles, CA United States; 3 Department of Family Medicine David Geffen School of Medicine Los Angeles, CA United States; 4 Department of Pediatrics David Geffen School of Medicine Los Angeles, CA United States

**Keywords:** patient portal, safety net, health disparities, digital divide

## Abstract

Despite the implementation of internet patient portals into the safety net after the introduction of the Affordable Care Act in the United States, little attention has been paid to the process of engaging vulnerable patients into these portals. The portal is a health technology tool that was developed with a mainstream, English-speaking audience in mind. Thus, there are valid concerns that such technologies will actually exacerbate health care disparities, conferring further advantages to the already advantaged. In this paper, we describe a framework for portal engagement (awareness, registration, and use) among safety net patients. We incorporate the experiences in the Los Angeles County Department of Health Services to illustrate important contextual factors for portal outreach in our safety net. Finally, we discuss considerations for moving forward with health technology in the safety net as the next version of patient portals are being developed.

## Introduction: A Patient Portal Arrives to the Safety Net

Online patient health portals, also known as “patient portals” or “portals,” are two-way communication systems that are tethered to a patient’s electronic health record (EHR) [1]. Portals allow patients to manage many aspects of their health care from the convenience of their internet-connected device. Some studies have found that use of patient portals may improve quality of care and health outcomes, particularly for patients with chronic conditions such as diabetes mellitus [1-10].

Largely driven by the financial incentives of the Health Information Technology for Economic and Clinical Health Meaningful Use program (as part of the 2014 US federal health care reform), patient portals have rapidly expanded [2,11-13]. In the last few years, many safety net systems—which are health systems that provide a significant level of care to minority, low-income, limited English proficient (LEP), and other vulnerable patients—have begun implementing patient portals. This innovation has given safety net health systems a new mechanism to directly share information and communicate with patients online. Since many vulnerable patients face barriers to in-person visits (such as taking unpaid time off from needed work), this telemedicine mechanism has potential for enhancing their care. Most notably, the coronavirus-19 (COVID-19) disease pandemic forced health systems to scale back on physical patient visits, and in-person patient education and engagement dramatically. With no established telemedicine workflows in place, this situation can exacerbate the health disparities for these patients who are already at higher risk of poor disease management outcomes. Recent events have therefore highlighted the need to prioritize the integral role of the patient portal for care delivery in the Los Angeles safety net, and safety nets across the country.

Much of the prior literature related to patient portals and low-income populations has focused on the barriers posed by the digital divide; that is, the fact that many vulnerable populations lack the digital access, capacity, and interest to use a portal [14-28]. However, prior national studies suggest that the racial/ethnic and socioeconomic digital divide is shrinking, and that there are no racial/ethnic differences among people accessing the internet via mobile phones (about 60% of US adults) [28,29]. Furthermore, new data corroborate and expand upon prior work showing a high level of interest in portals among low-income, LEP, Medicaid, and public hospital patients [30-34]. Thus, the aforementioned barriers to the use of portals by safety net populations may be diminishing, and this population may be increasingly ready and eager to use the patient portal.

Despite these findings, little attention has been paid to the *process of engaging vulnerable patients* into an online health portal. This is especially problematic as the portal is a health technology tool that was developed with a mainstream, English-speaking audience in mind [13,35,36]. There are valid concerns that such technologies, including the patient portal, will actually exacerbate health care disparities, conferring “further advantages to the already advantaged,” a tenet of the Inverse Care Law [26].

As safety net health systems continue to implement patient portals [37], important questions remain about the factors that influence safety net portal registration and use, and the portal education strategies that will be effective among these vulnerable patients [13,15,35,38-40]. Health systems that are developing portals for vulnerable populations might benefit from an underlying framework that takes into consideration the unique needs of these populations.

In this paper, we describe our portal engagement process (awareness, registration, and use) for safety net patients. We then incorporate the experiences in the Los Angeles County Department of Health Services (LAC DHS) between 2015 and 2019 to illustrate important contextual factors in our safety net’s portal development. Finally, we discuss considerations for moving forward with health technology in the safety net as the next version of patient portals are being developed.

## Development of a Framework for Portal Engagement Among Vulnerable Patients in the LAC DHS

### Overview of Formative Findings

**Figure 1:**
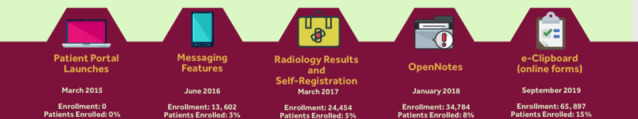
Los Angeles County Department of Health Services portal features timeline and percentage of empaneled primary care patients enrolled at each stage.

The LAC DHS, as the second largest municipal health safety net system in the United States, launched its English-Spanish patient portal in March 2015, with one of the few bilingual interfaces in the nation. The LAC DHS serves 600,000 unique patients each year, with 400,000 patients empaneled to its primary care clinics. Over half of the LAC DHS population is LEP, with the majority of these patients being Spanish speakers. At its inception, patients were able to view lab results, medication lists, and vital signs; additional features were added progressively. Figure 1 outlines the timing of various portal features in the LAC DHS *MyWellness* patient portal and accompanying portal registration data.

Many of these portal developments/improvements stemmed from patient input that we received on the frontlines. From January to March 2016, the LAC DHS performed a system-wide quality improvement internal survey of patients waiting in line for medical records. Patients were asked to fill out a paper or tablet survey to self-describe any internet access (including public and private access), knowledge about the *MyWellness* patient portal, and interest in health information on the *MyWellness* patient portal. We systematically randomly surveyed almost 200 patients, 73.0% (n=146) of whom reported having access to the internet. Only 20.0% (n=40) of patients were aware of the patient portal, and 45.0% (n=90) of those surveyed indicated interest in learning more about the portal.

**Figure 2:**
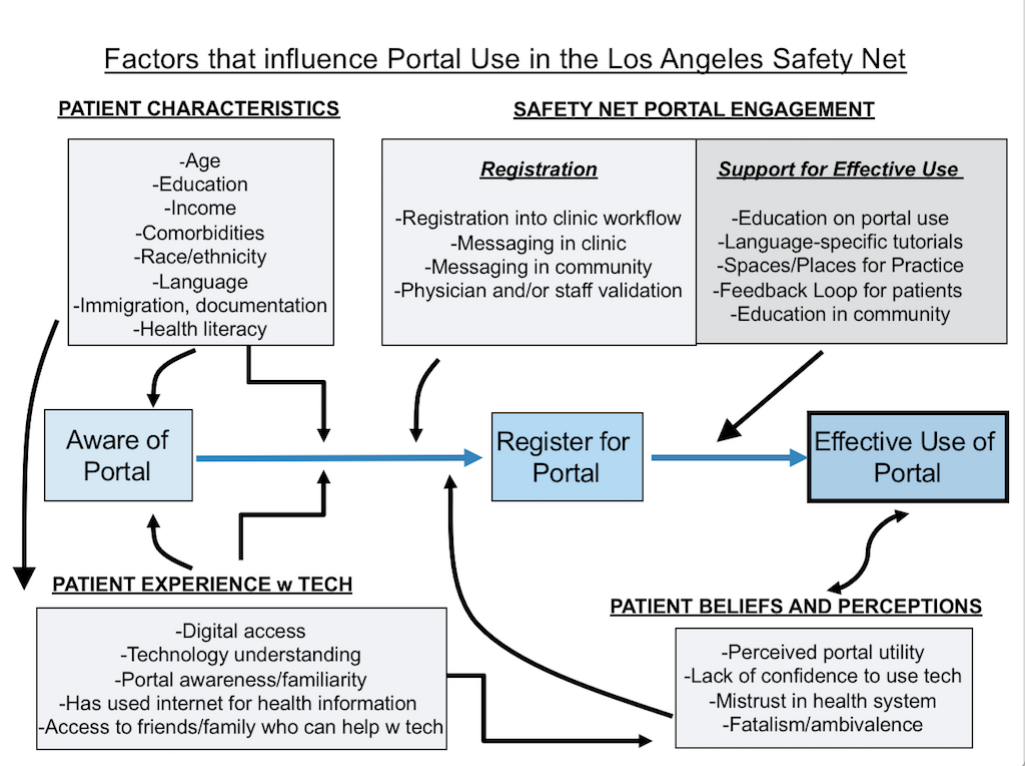
Implementation framework for portal registration and use among safety net patients.

We conducted focus groups in the summer of 2017 among LAC DHS patients with chronic conditions to better understand perceptions of the portal and to obtain patient-centered recommendations for implementation [41]. Important themes from these focus groups were that we needed to provide dedicated patient guidance for both portal enrollment and portal navigation. One of the LAC DHS sites received a Catalyst human-centered design grant through the Center for Care Innovations to better understand the patient process and experience around trying to register for the patient portal. This formative work culminated in the development of a novel framework to outline factors affecting portal registration and use in the safety net, which is partly based on the technology acceptance model [42]. As shown in Figure 2, the framework outlines factors under the domains of *patient characteristics, patient experience with technology, patient beliefs and perceptions, and engagement initiatives by safety net health systems* that will impact (1) patient awareness of a patient portal, leading to (2) patient registration, and ultimately result in (3) sustained use of the portal by patients.

Our formative findings and framework coincide with some of the conclusions provided by Grossman et al [43] in 2019. After reviewing over 100 studies about patient portal use among vulnerable populations, they found that individually focused interventions had the most evidence for increasing use in vulnerable populations. They recommended that research should “move beyond identifying disparities to systematically addressing them at multiple levels” for these patients [43]. In that vein, our work over the last 5 years has led to three actionable steps in facilitating portal uptake among our patients, which we now recommend as a model to other safety nets and EHR companies serving these patient populations:

### Step 1: Remove Unnecessary Patient Process Barriers in Enrolling for the Portal

Recent studies have provided recommendations on patient- and system-level interventions to increase registration and use of the portal (ie, opt-out enrollment strategies, patient portal training) [43]. However, many of these recommendations will depend on the specific restrictions and parameters of the EHR vendor. For our patient population, the LAC DHS originally required an email address and a social security number (SSN) as a unique patient identifier (to receive a patient portal activation code). Privacy is paramount as it pertains to health data and security; however, authentication that requires an email address for portal enrollment can widen digital disparities to data access in the safety net. An acceptable alternative could be sending the activation code via a cell phone number (although this does not overcome the barrier that patients of low socioeconomic status may share phones). At one of our largest LAC DHS sites, patients between the ages of 18-64 have 75%-87% cell phone ownership and 49% of the patients aged>65 years report cell phone ownership. Authentication processes that require an SSN in safety net populations are especially problematic, given that many immigrant patients may lack this information or even fear a process asking for it, owing to their (or their family members’) documentation status. In response to these issues, we worked with our vendor to eliminate these steps and replace them with acceptable enrollment alternatives (eg, self-registration, medical record number identifiers).

*To improve equity to data access, we suggest that EHR vendors improve the ease of enrollment and give patients options for multifactor authentication that do not require an email address or secure/stigmatized information.*

### Step 2: Rethink Portal Engagement With a Patient-Centered Approach

The creation of LAC DHS Patient Portal Summits have brought together leadership, patient advocate groups, front desk staff, and clinicians to uniformly design engagement materials and staff incentive programs that best advance portal registration among our patients (even in low-resource settings). With support from a 2018 Center for Care Innovation’s Catalyst grant, we created portal engagement materials that could eventually be administered by any LAC DHS staff member. Patients, nursing, staff, and physicians from all LAC DHS sites provided input over the course of 18 months. To date, this collaboration has resulted in a training curriculum for LAC DHS staff on portal engagement and engagement videos featuring the portal, developed by (and showing) patient and staff members and displayed in waiting rooms across our system in multiple languages [42,44]. From November 2018 to January 2019, a patient portal enrollment competition took place to further engage frontline staff in enrolling and engaging patients in the portal.

*Based on concepts that were prominent in our own formative work with patients,* *we suggest that safety net systems improve their portal messaging approaches to focus on: (a) validation of the portal by health care workers, (b) messaging about the portal that is useful to a patient’s daily life and relevant to their personal health [45], and (c) educational scripts that incorporate family/community members.*

### Step 3: Partner With the EHR Vendor to Focus on an Appropriate User Interface

Our data show that the majority of LAC DHS patients who access the portal do so via their mobile devices (70%), whereas portals from nonsafety-net patients tend to more frequently be accessed via a desktop [28]. Pew Research surveys on smartphone use describe a phenomenon of “smartphone dependence” among low-income and minority populations [28]. Cable internet is increasingly expensive, and low-income communities increasingly depend on smartphone internet access for their overall online access. This is particularly important to keep in mind as patients are accessing health data. At the LAC DHS, we recognize that our internet strategies must be “mobile first.” Accordingly, true mobile-friendly experiences became a ripe area for co-design with our patients, health system, and EHR vendors. In addition to prioritizing the improvement of our mobile app version of the *MyWellness* portal over the desktop app, and ensuring that *all* portal features are available on the mobile version, we are also working with our EHR vendor on the following aspects: improving usability of the portal website for non-English speakers, making messaging more obvious on the website, improving patient education options to “learn more” about a lab or health condition, using multiple languages, creating a patient virtual feedback group, and forming a staff “super-user and champions” group to report back to our EHR vendor regarding usability concerns for our patients.

Another limitation has been the unwillingness of large portal vendors to allow the use of images from the portal in creation of training materials. This is a simple barrier that must be addressed, and just another example of how health systems should better partner with their EHR vendors on relevant patient portal operations and research [46]. We recognize that improving the user interface and usability will be a key challenge for this partnership, although there are published models that can help us better understand how users interact with this technology and the resources that may be necessary to support its use [47]. Portals are not alone in this limitation, as most digital health apps and platforms do not meet basic health literacy or language standards. Indeed, there are no clear standards for digital engagement in health care, and this gap must be addressed if the portal is to become completely accessible for diverse populations.

*Despite very specific standards for health literacy of written materials, this has not been translated to digital tools. Therefore, we recommend more transparency and inclusion around testing strategies to ensure that safety net populations are active participants in these testing phases.*

## Patient Portal 2.0: Reimagining the Next Generation of Portals for Improved Accessibility to Safety Net Patients

### Key Questions

If health technology is destined to serve even the most vulnerable patients, medical informatics research must answer the following questions: How can portals be made more accessible from the patient’s perspective? What do safety net patients need from patient portals? By focusing on patients from vulnerable backgrounds, the safety net setting becomes a real-life laboratory for developing portals that will improve portal accessibility for all populations. We highlight the following patient-centered points (findings from our formative work) in reimagining the next version of portals.

### Connect to the Safety Net Patient’s “Home” Team

Outside of the health care setting, there is already a “team” surrounding many of our patients, made up of family members and friends who serve as caregivers and trusted confidants for health decisions. Therefore we need to make it easier to connect digitally with these trusted team members (in addition to the individual patient) if the patient portal is to be used as a primary health management tool moving forward.

To first address this, there must be better options around “level of access” to patient data through proxy relationships, especially when patient privacy and security remain a top level of concern among safety net patients. Our LAC DHS physicians have reported the need to provide limited portal views of “sensitive” information (eg, HIV results, intravenous drug use history) for patients who rely on family or friends as informal or formal caregivers. The option to share the portal with the patient’s team may be foregone in some cases because of the current “all-or-nothing” access approach to health information via a proxy login. One solution to this dilemma is to allow patients the ability to choose what level of proxy access a caregiver/family member will have. In reality, this is a feature that should be available to all patients, and will particularly resonate with adolescent and geriatric populations, and for patients with disabilities in other health care settings.

### Create a Virtual Home for Patient-Centered Care

What if the portal 2.0 were *more* than a data repository and messaging/scheduling hub? Patients are starting to fill out forms online through the patient portal, and such patient-generated data should be helpful to health care systems for tailored patient care and improvements to the system. What if the portal could also suggest content and experiences for patients? For example, a patient recently diagnosed with heart failure could receive suggestions from the portal for post-discharge follow-up videos, and health systems could track these engagement metrics. The portal could also push information to the patient about heart failure options for disease monitoring, such as online weight logs or a wireless scale. Patients with prediabetes who want support for lifestyle modification could be directed to locations for free or low-cost exercise programs in recreation centers or parks. Patients and providers have already suggested a desire for a portal that can collect patient-reported outcome metrics and deliver personalized feedback [48].

Currently, health systems are reactive; that is, we often wait for patients to approach our team with questions before we address health issues and socioemotional concerns. However, if a patient fills out a “Know Me” questionnaire and sets personal weight loss goals, the portal might be able to suggest culturally tailored nutrition and physical activity community resources for the patient based on their profile, even before visiting the clinic. As another example, if housing instability is listed as a problem in the EHR, the portal should potentially be able to push local resource notifications for housing or legal aid services. When “food insecurity” is recognized and coded in the EHR, could the portal then push notifications about food resources that are tailored to the patient’s home address or the geolocation on their phone? The safety net can help us re-envision the portal as a place where: (1) patients can be connected with social resources based on their needs, rooted in the social determinants of health; (2) patients can “check in” on health indicators, track the progression of personal indicators and goals; and (3) access culturally tailored and language-appropriate videos, podcasts, and written materials based on the patients’ underlying health conditions.

### Moving Beyond Meaningful Use

To achieve this vision of the patient portal, we must grade new portals based on metrics that move beyond meaningful use*,* which have previously only focused on patients’ log-in, download, and exchange of data/messages with their health care team. EHR vendors are starting to understand the need to assist patients with self-management by exploring workflows to help patients fill out forms online to monitor chronic conditions, creating online interfaces for patients to log blood sugar and blood pressure levels, working to integrate devices such as smartwatches and connected devices (ie, wireless glucometers, blood pressure cuffs, weight scales), incorporating care manager and patient goals into their platforms, and integrating online platforms for community-based resources such as food banks, transportation assistance, and housing into the health record.

## Summary: How Patient Portals Will Meet the Needs of Safety Net Patients

Safety net health systems provide health care for our most medically and socially fragile patients: populations that include patients with multiple morbid conditions, LEP, cognitive impairment, high-risk perinatal needs, physical and mental disabilities, low literacy, homelessness, substance use, justice system–affected, and a broad range of immigrant and refugee communities. Safety nets are the ideal places to develop and refine the next iterations of the EHR and the patient portal. Because the portal is a “gateway” to the use of other digital health interventions, it is also an avenue for intervention research and a modality for better understanding of what vulnerable patients need to more effectively interface with health technology.

A limitation of this paper is that we did not delve into the continual barriers that sustain the digital divide (having access to reliable internet, limited devices and data/storage, and patients’ digital literacy). Although the digital divide seems to be shrinking, these recommendations should also be tempered with the knowledge that extreme disparities in internet access continue to exist for low-income populations, especially in certain areas of the country [49]. All of these systemic and patient-centered barriers should be addressed in a version of patient portal development that is more inclusive. To make this tool work for our most vulnerable populations, moving forward, we need to take intentional steps to ensure that the patient portal can be effectively and efficiently deployed in health systems that serve these high-risk patients. To achieve these goals, we must (1) remove unnecessary patient process barriers in enrolling for the portal; (2) redesign engagement materials with a patient-centered approach; (3) partner with EHR vendors to focus on the user interface and usability from a safety net patient perspective; (4) engage trusted family members and caregivers to create a flexible, patient-friendly mechanism for proxy access; (5) create a virtual home for patient-centered care that includes addressing social determinants, preventive care, and chronic care; and (6) redefine the metrics of portal success, as seen in the safety net.

Finally, we would be remiss in not emphasizing that health systems around the country are quickly developing remote strategies to reach out to patients for health management resources and education as in-person services shrink, secondary to the COVID-19 pandemic, and that the patient portal is an integral part of this outreach plan. Thus, redesigning the patient portal so that it effectively reaches and impacts our most vulnerable patients is an important step to improve health care access for the entire US population. These uncertain and challenging times in our history are an opportunity to significantly move the needle in digital health, and create a patient portal that works for even the most vulnerable patients in this new era of healthcare— making sure that no patient is left behind.

## Abbreviations

COVID-19: coronavirus disease 2019
EHR: electronic health record
LAC DHS: Los Angeles County Department of Health Services
LEP: limited English proficient
SSN: social security number
